# Objectification of intracochlear electrocochleography using machine learning

**DOI:** 10.3389/fneur.2022.943816

**Published:** 2022-08-29

**Authors:** Klaus Schuerch, Wilhelm Wimmer, Adrian Dalbert, Christian Rummel, Marco Caversaccio, Georgios Mantokoudis, Stefan Weder

**Affiliations:** ^1^Department of ENT, Head and Neck Surgery, Inselspital, Bern University Hospital, University of Bern, Bern, Switzerland; ^2^Hearing Research Laboratory, ARTORG Center for Biomedical Engineering Research, University of Bern, Bern, Switzerland; ^3^Department of Otorhinolaryngology, Head and Neck Surgery, University Hospital Zurich, University of Zurich, Zurich, Switzerland; ^4^Support Center for Advanced Neuroimaging (SCAN), University Institute for Diagnostic and Interventional Neuroradiology, Inselspital, Bern University Hospital, University of Bern, Bern, Switzerland

**Keywords:** ECochG, signal processing, deep learning, Hotelling's T^2^, correlation analysis, residual hearing, electroacoustic stimulation, cochlear implant

## Abstract

**Introduction:**

Electrocochleography (ECochG) measures inner ear potentials in response to acoustic stimulation. In patients with cochlear implant (CI), the technique is increasingly used to monitor residual inner ear function. So far, when analyzing ECochG potentials, the visual assessment has been the gold standard. However, visual assessment requires a high level of experience to interpret the signals. Furthermore, expert-dependent assessment leads to inconsistency and a lack of reproducibility. The aim of this study was to automate and objectify the analysis of cochlear microphonic (CM) signals in ECochG recordings.

**Methods:**

Prospective cohort study including 41 implanted ears with residual hearing. We measured ECochG potentials at four different electrodes and only at stable electrode positions (after full insertion or postoperatively). When stimulating acoustically, depending on the individual residual hearing, we used three different intensity levels of pure tones (i.e., supra-, near-, and sub-threshold stimulation; 250–2,000 Hz). Our aim was to obtain ECochG potentials with differing SNRs. To objectify the detection of CM signals, we compared three different methods: correlation analysis, Hotelling's T^2^ test, and deep learning. We benchmarked these methods against the visual analysis of three ECochG experts.

**Results:**

For the visual analysis of ECochG recordings, the Fleiss' kappa value demonstrated a substantial to almost perfect agreement among the three examiners. We used the labels as ground truth to train our objectification methods. Thereby, the deep learning algorithm performed best (area under curve = 0.97, accuracy = 0.92), closely followed by Hotelling's T^2^ test. The correlation method slightly underperformed due to its susceptibility to noise interference.

**Conclusions:**

Objectification of ECochG signals is possible with the presented methods. Deep learning and Hotelling's T^2^ methods achieved excellent discrimination performance. Objective automatic analysis of CM signals enables standardized, fast, accurate, and examiner-independent evaluation of ECochG measurements.

## 1. Introduction

Electrocochleography (ECochG) measures electrical potentials generated by the inner ear in response to acoustic stimulation. In patients with cochlear implant (CI), using the implanted electrode, these potentials can be picked up directly from the inner ear. The technique is increasingly used to monitor the inner ear function during and after implantation. Research groups were able to correlate changes in the ECochG signal with traumatic events during implantation (1–6).

In order to assess ECochG potentials (either intra or postoperatively), the analysis is most commonly performed by visual inspection, which is currently the gold standard. Therefore, the interpretation is heavily relying on the expertise of the examiner. This entails several problems: i) a high level of experience is needed to interpret the signals correctly. Thus, inexperienced clinicians and researchers are unable to exploit the technique; ii) the examiner determines whether or not an ECochG response is present, which may result in a lack of reproducibility; iii) longitudinal comparisons are hampered as the assessment is not absolutely identical. iv) research groups use different types of analysis, which makes the comparability of clinical findings and study results difficult or impossible (4, 7–12); v) due to the inconsistent assessment, patients with a poor signal-to-noise ratio (SNR) are often not reported. However, in order to draw correct conclusions, all measurements should be reported (13, 14); and vi) the analysis of ECochG signals is complex, which makes immediate judgment difficult. This is, of course, a prerequisite when an instant assessment is required (e.g., in the operating theater).

ECochG itself is an umbrella term for different electrophysiological signal components of the inner ear (i.e., the cochlear microphonic, CM, the auditory neurophonic, ANN, the compound action potential, CAP, the summating potential, SP). These signal components can be highlighted by measurements with different acoustic polarities (condensation, CON and rarefaction, RAR). The difference potential (DIF) is calculated by subtracting the CON and RAR polarities. The DIF response mainly represents the CM signal (15). In addition, the sum highlights the summating potential (SUM), which mainly represents the ANN (16). However, CM and ANN potentials cannot be isolated, especially at high stimulation levels and low frequencies (17). In intra and postoperative recordings, most commonly the CM/DIF signal is used as it is the largest and most robust signal component (18). For this reason, in this article, we will limit the analysis to the CM/DIF signal. Even though the CM/DIF signal is the strongest potential, there are some things to keep in mind. The amplitude of the signal is in the microvolt range and varies greatly between individuals. While certain patients show large amplitudes, in others, the potentials are very small, resulting in a poor SNR. Furthermore, the morphology and latency of the CM/DIF signal might vary significantly depending on the remaining intact hair cells (19–21). These factors (i.e., poor SNR, different wave morphology) must be taken into account when analyzing ECochG potentials.

For the reasons given above, an automated and objective evaluation would be highly desirable. This would standardize and significantly simplify the analysis of the signals and make it independent of the examiner. For ECochG signals, an approach using Fast Fourier Transform (FFT) has been proposed (18, 22–24). However, this method is not always applicable, especially for short signals, since they do not have a stationary period and adjacent frequencies cannot be accurately distinguished. For other electrophysiological signals, objectified analyses have become established in clinical practice. For example, for auditory brainstem responses (ABR), correlation analysis is used (25, 26). In the evaluation of cortical auditory evoked potentials (CAEP), Hotelling's T^2^ test has yielded a sensitivity at least comparable to that of visual inspection (27–29). In other medical disciplines (i.e., identification of cardiac arrhythmias in electrocardiograms, ECGs), deep learning (DL) strategies could be successfully implemented (30–33).

The aim of this study was to automate and objectify the analysis of CM/DIF signals in ECochG recordings. The employed method should i) be comparable to visual analysis, (ii) allow the interpretation of intra- and postoperative ECochG signals by clinicians and researchers who do not have much experience in the field, (iii) allow immediate feedback, (iv) should be replicable by other clinical and research centers, (v) allow reproducible comparison of longitudinal data (since the same analysis is performed).

## 2. Materials and methods

This prospective cohort study was conducted in accordance with the Declaration of Helsinki and was approved by the local institutional review board (KEK-BE 2016-00887 and 2019-01578). All participants gave written informed consent before participation.

### 2.1. ECochG data

We performed ECochG measurements in 36 subjects (*n* = 41 ears). All subjects used a Med-El implant (MED-EL, Austria). Pure tone audiograms were performed in a certified acoustic chamber with a clinical audiometer (Interacoustics, Denmark). Hearing thresholds were collected either immediately preoperatively or, in the case of postoperative measurements, on the same day as the ECochG measurement. We obtained pure tone air conduction hearing thresholds in dB hearing level (HL) at 125, 250, 500, 750, 1,000, 1,500, 2,000, and 4,000 Hz using either headphones or plug-in earphones. Pure tone averages (PTAs) were calculated as the mean hearing threshold at 125, 250, 500, and 1,000 Hz. PTAs and patient demographics are shown in Table 1.

**Table 1:** Demographic of included subjects.

**Subject ID**	**Gender**	**Age (years)**	**Side**	**Etiology**	**Electrode**	**ToM (month)**	**PTA (dB HL)**
io 1	M	49	L	Meningitis	Flex 28	io	52.5
io 2	M	69	L	Progressive HL	Flex 28	io	58.8
io 4	F	45	L	Progressive HL	Flex 28	io	93.8
io 5	F	60	L	Progressive HL	Flex 24	io	66.3
io 6	M	51	R	Progressive HL	Flex 28	io	60.0
io 7	M	75	R	Progressive HL	Flex 28	io	52.5
io 8	F	77	L	Progressive HL	Flex 28	io	75.0
io 9	M	36	R	Congential genetic	Flex 26	io	48.8
io 10	M	71	R	Progressive HL	Flex 28	io	71.3
io 11	F	70	L	Progressive HL	Flex 28	io	50.0
io 12	F	27	R	Congential genetic	Flex 28	io	62.5
io 13	M	66	R	Meniere's disease	Flex 28	io	72.5
io 14	F	53	L	Progressive HL	Flex 28	io	78.8
io 15	M	59	R	Progressive HL	Flex 28	io	48.8
io 16	F	78	L	Progressive HL	Flex 28	io	86.3
io 17	F	28	R	Progressive HL	Flex 26	io	33.8
io 18	M	86	L	Progressive HL	Flex 26	io	91.3
io 19	M	21	R	Progressive HL	Flex 28	io	78.8
io 20	F	61	R	Sudden HL	Flex 28	io	81.3
io 23	M	59	L	Progressive HL	Flex 28	io	77.5
io 24	F	37	L	Sudden HL	Flex 26	io	83.8
po 0	F	60	R	Progressive HL	Flex 28	10	68.8
po 1	M	73	R	Progressive HL	Flex 28	17	110.0
po 2	M	75	L	Progressive HL	Flex 24	46	66.3
po 3	M	80	L	Congential genetic	Flex 28	9	85.0
po 4	F	27	R	Congential genetic	Flex 28	20	101.3
po 5	F	66	R	Progressive HL	Flex 28	28	92.5
po 6	F	73	R	Meniere's disease	Flex 28	78	90.0
po 7	M	82	L	Progressive HL	Flex 28	75	113.8
po 8	F	25	R	Congential genetic	Flex 28	57	85.0
po 9	F	43	R	Progressive HL	Flex 28	22	83.8
po 10	F	60	R	Progressive HL	Flex 24	13	97.5
po 11	F	73	L	Progressive HL	Flex 28	70	100.0
po 12	M	50	R	Meningitis	Flex 28	11	81.3
po 13	F	68	L	Progressive HL	Flex 28	22	93.8
po 14	F	52	R	Congential genetic	Flex 24	174	95.0
po 15	M	50	L	Meningitis	Flex 28	6	75.0
po 16	M	66	R	Meniere's disease	Flex 28	7	106.3
po 17	M	56	R	Sudden HL	Flex 28	11	91.3
po 18	M	75	R	Progressive HL	Flex 28	70	96.3
po 19	F	63	R	Progressive HL	Flex 24	131	91.3
Mean		58.4				43.9	79.2

PTA, pure tone average; HL, hearing loss; ToM, time of measurement in months after implantation; io, intraoperative; po, postoperative.

We recorded ECochG potentials using the Maestro Software (version 8.03 AS and 9.03 AS, MED-EL, Austria). The system setup was identical to our previous study (10). We measured ECochG potentials at electrodes 1, 4, 7, and 10 (with electrode 1 at the tip) and only at a stable electrode position (i.e., either intraoperatively after completed electrode insertion or in a postoperative setting). When stimulating, depending on the individual hearing threshold, we used three different intensity levels: supra-threshold level (5 dB below discomfort level), near-threshold level (10 dB above hearing threshold), and sub-threshold level (10 dB below hearing threshold). Thereby, the acoustic amplitude level was restricted as shown in Table 2. Our aim was that not all stimulations would elicit an ECochG response and that, depending on the stimulation level, the SNR was different. As an acoustic stimulus, we used pure tones with settings shown in Table 2. ECochG potentials were recorded with two polarities (i.e., CON, and RAR). For each ECochG response, we recorded 100 epochs per polarity. The two polarities were subtracted to form the CM/DIF signal.

**Table 2:** Settings for acoustic stimulation and maximum possible acoustic stimulation level (maximum amplitude).

**Frequency**	**Stimulus**	**Recording**	**Measurement**	**Maximum**
**(Hz)**	**duration**	**delay**	**window**	**amplitude**
	**(ms)**	**(ms)**	**(ms)**	**(dB HL)**
250	12	1	19.1	109
500	8	1	9.6	115
750	6.67	1	9.6	123
1,000	5	1	8.0	122
1,500	4	1	8.0	122
2,000	3	1	6.5	122

### 2.2. Preprocessing of ECochG signals

As preprocessing, we used the following steps: i) if present, removal of stitching artifacts, ii) application of a Gaussian weighted averaging method to increase the SNR and exclude uncorrelated epochs from further analysis, and iii) a 2nd order, forward-backward filtered Butterworth bandpass filter (cutoff frequencies 10 Hz / 5 kHz for visual analysis, and 100 Hz / 5 kHz for objective evaluation methods). To increase the SNR in our ECochG recordings, we calculated the Gaussian weighted epochs *S*_*GE*(*i*)_ as described by Davila et al. (34) and Kumaragamage et al. (35). We used the following equation:


$$\begin{array}{l} S_{GE\left(i\right)}=\overset{2}{\underset{l=-2}{\sum }}\left(e^{-\left[0.5{\left(\frac{l}{\sigma \cdot \left(5-1\right)/2}\right)}^{2}\right]}\cdot S_{E\left(i+l\right)}\right) \end{array}$$

whereas, *l* is the index number, starting from –2 to 2 that accounts for five epochs *S*_*E*_ averaged under the Gaussian window, and *i* is the index number of the epochs in *S*_*E*_. The SD of the Gaussian window σ was set to 0.4. Each Gaussian weighted epoch *S*_*GE*(*i*)_ was then correlated with the mean of all epochs *S*_*approx*_. *S*_*GE*(*i*)_ with a correlation less than –0.2 were excluded to form the final ECochG response *S*. If more than 10% of epochs had to be removed, only the 10 worst correlated were discarded. Finally, we calculated the SNR using the +/- averaging method (36).

### 2.3. Visual analysis

ECochG data were visually analyzed by three examiners with extensive experience in the field. The goal was to have a labeled data set that was used i) to train and test the objective algorithms, and ii) to obtain a benchmark for evaluating the accuracy, specificity, and sensitivity of the objective detection methods. Using Labelbox (37), the data were presented to the examiners as a subplot with six individual graphs representing i) the DIF response, ii) the SUM response, iii) the CON and RAR responses, and iv-vi) their individual FFT traces (an example is shown in the Supplementary material). Each examiner had to assess 4133 ECochGs with the question if a CM/DIF response was present or not (dichotomous question). Thereby, we used a blinded design in which the investigators did not discuss the assessment to avoid bias in the individual assessment. Signals classified as CM/DIF response by two examiners (and noise by one examiner) were presented a second time to all three examiners (to minimize volatility errors). Only ECochG signals that were finally considered valid responses by all three investigators were classified as responses. These were used as ground truth for the objective classification. We used Fleiss' kappa to compare the raters. Fleiss' kappa is a measure of agreement between multiple raters in classifying items (38).

### 2.4. Objective detection methods

We included the following objective detection methods: i) Hotelling's T^2^ test, ii) correlation analysis, and iii) a DL convolutional neural network (CNN). To train and evaluate our objective analysis, we benchmarked these methods against the visual analysis of the three experts.

The dataset was divided into two parts: 70% for training and 30% for testing purposes. We used the training subset to train and validate the models. For training, both features (ECochG signals) and labels (ground truth determined by the examiners) were provided. The test set was used to evaluate the performance of the model. Here, only features were provided. The predictions of the model were then compared to the labels.

#### 2.4.1. Hotelling's T^2^ test

Based on Hotelling's T^2^ method described by Golding et al. and Chesnaye et al. for objective detection of CAEP signals, we adapted the method to ECochG signals (27, 29). The Hotelling's T^2^ test for one sample is a multivariate extension of the Student's *t*-test (39, 40). With Hotelling's T^2^ test, we can test the null hypothesis (H0) whether Q features are statistically different from Q hypothesized values.

In our case, the ECochG recordings were the features and the hypothesized values were noise. The ECochG recordings were divided into Q windows along the time axis called 'time-voltage-means' (TVMs). The mean value was taken from each Q-window, resulting in the following N × Q voltage matrix V:


$$\begin{array}{l} V=\left[\begin{array}{ccc} v_{11} & \ldots & v_{1Q}\\ \vdots & \ddots & \vdots \\ v_{N1} & \ldots & v_{NQ} \end{array}\right] \end{array}$$

**Figure 1:**
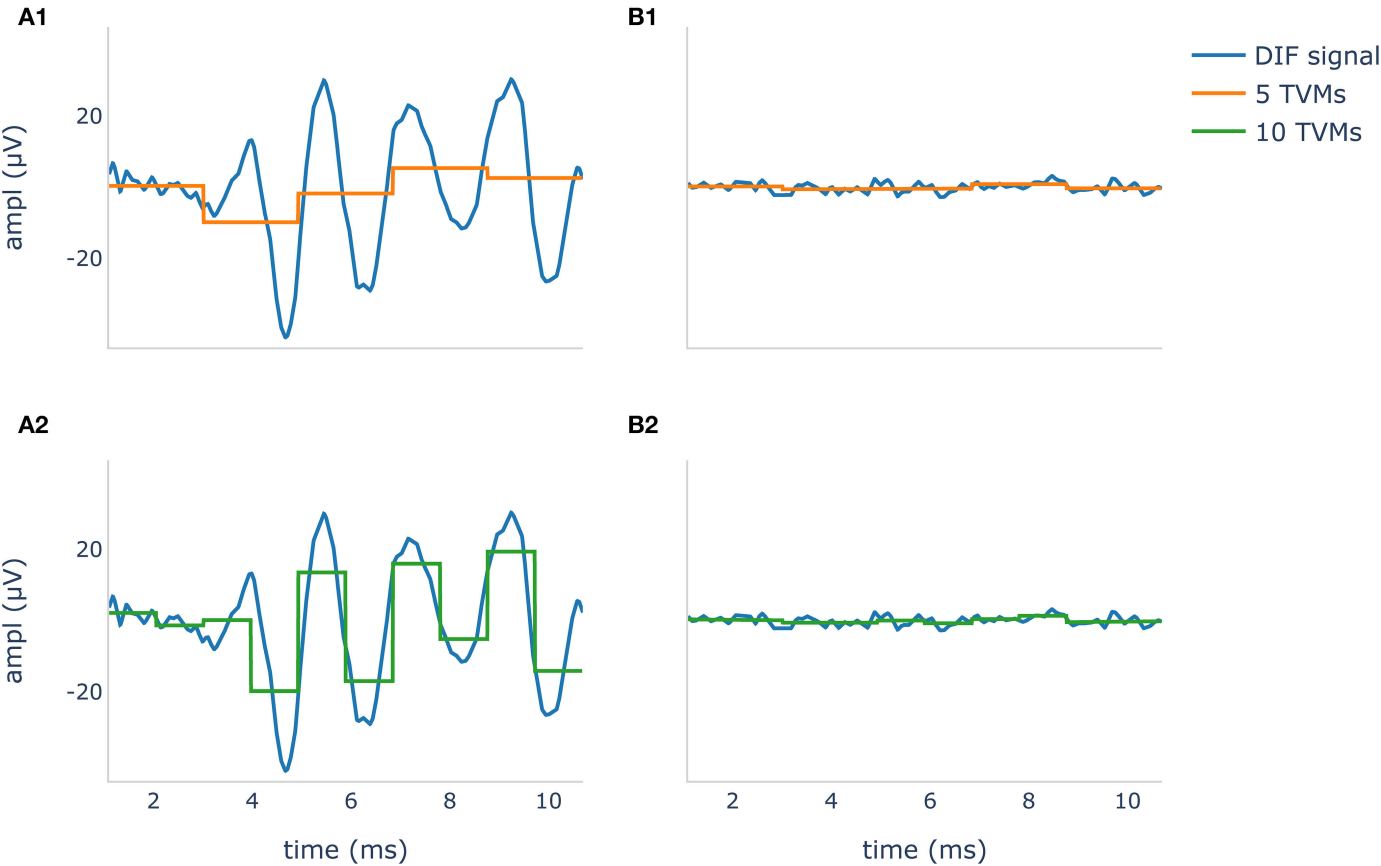
Difference potential (DIF) curves in blue show a recognizable CM/DIF signal **(A1,A2)** and noise with no visible CM/DIF component (B1, B2) in response to a 500 Hz stimulus. The orange curve in **(A1,B1)** shows 5 time-voltage-means (TVMs), and the green curve in **(A2,B2)** shows 10 TVMs used to calculate Hotelling's T^2^ test. It is evident that in this example, an increase of the TMVs leads to better mapping of the CM/DIF signal with higher accuracy.

Where N was the number of epochs and v_*ij*_ the *j*^*th*^ voltage means from the *i*^*th*^ epoch. The corresponding hypothetical values (noise) were an array of size 1 × Q filled with zeros. The noise was zero because the expected mean value of an ECochG signal should be zero due to the bandpass filtering. The number of used TVMs resulted in a down sampling, illustrated in Figure 1.

We performed the calculations using a python (v 3.9.7) script and the *hotellings* function from the spm1d module (v 0.4) (41, 42). As significance level α, we used 0.01 to tune the number of voltage means Q for each acoustic stimulus frequency individually. The optimal number of TVMs for the Hotelling T^2^ test was calculated based on the maximum accuracy. For this purpose, the number of TVMs was successively increased in steps of five from 5 to 195 and the Hotelling's T^2^ test was calculated on the training set.

#### 2.4.2. Correlation analysis

Our correlation algorithm is based on the method of Wang et al. which explores the correlation of ABR signals (26). The correlation procedure relies on the repeatability of the similarity of two waveforms. The degree of similarity can be quantified by calculating the Pearson correlation coefficient. A positive correlation close to one reflects the presence of a response, while a zero correlation shows the absence of response (25).

**Figure 2:**
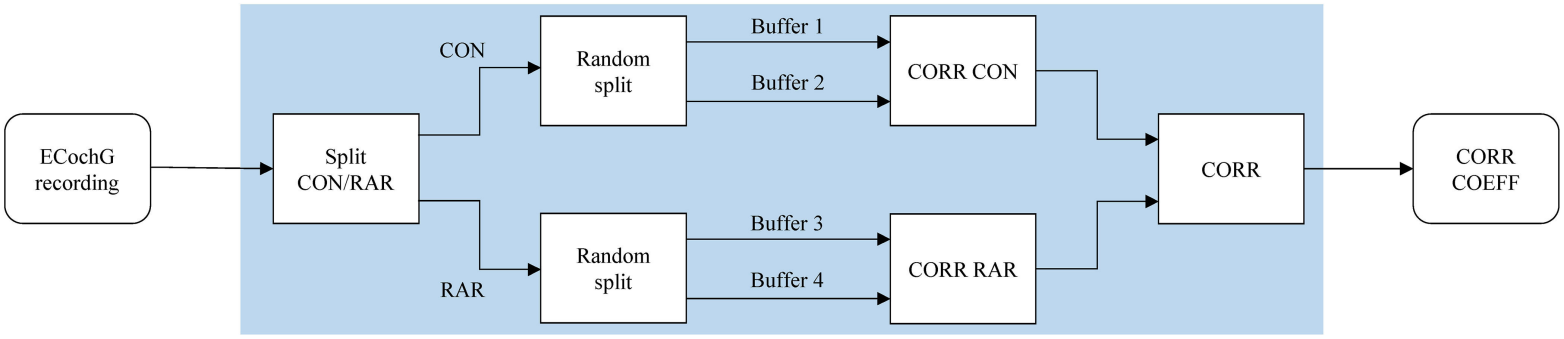
The correlation analysis handles CON and RAR recordings separately and proceeds as follows: (i) the ECochG recordings are divided into CON and RAR; (ii) CON and RAR are each divided into two randomly arranged buffers of the same size (Buffers 1–4, 50 epochs each); (iii) the Pearson correlation coefficients for CORR CON and CORR RAR are calculated from buffer 1 and 2 and buffer 3 and 4, respectively; (iv) CORR is calculated from the mean of CORR CON and CORR RAR. Since CORR depends on the subdivision of buffers, steps ii–iv (shaded area) are repeated 100 times and averaged to get the final correlation coefficient CORR COEFF. CON, condensation; RAR, rarefaction; CORR, correlation; COEFF, coefficient.

In our calculations, we treated the two polarities (CON/RAR) separately and finally averaged the correlation coefficients. The two polarities were separate, treated as they evolve inversely (which is caused by condensation and rarefaction phased acoustic stimuli). The procedure is shown in Figure 2. Finally, we fitted a logistic regression model based on the correlation coefficients.

#### 2.4.3. Deep learning

Our DL classification approach was based on the method used to automatically identify cardiac arrhythmia in ECG signals. Several DL approaches to cardiac arrhythmia detection have been proposed in the literature (30–33). Among them, time frequency scalograms using continuous wavelet transform (CWT) and AlexNet showed convincing results (32, 33). AlexNet is a large convolutional neural network (CNN) containing about 6,50,000 neurons and 60 million parameters. It consists of five convolutional layers, and three fully connected layers and is optimized for image classification (43).

**Figure 3:**
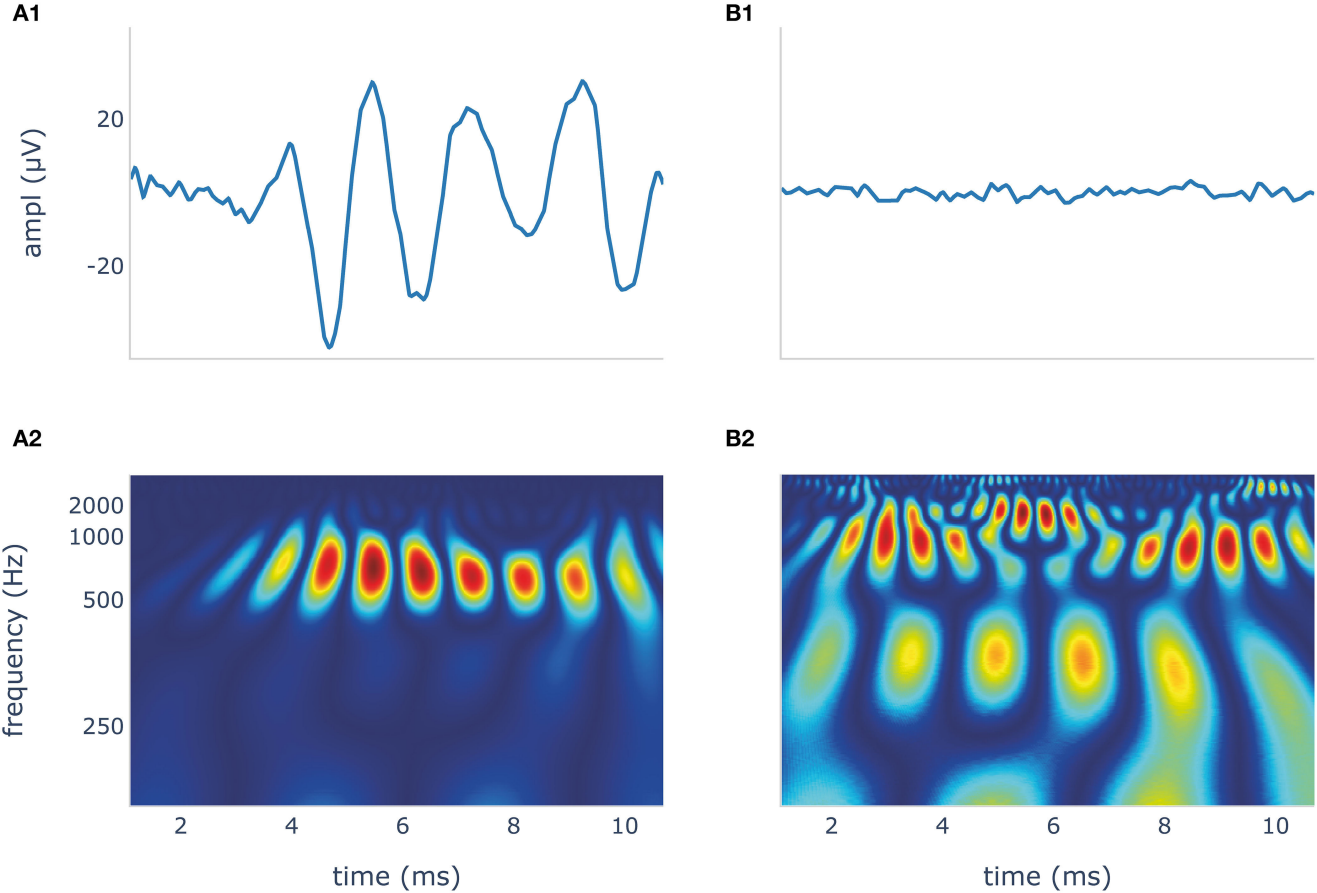
The blue DIF curves **(A1,B1)** show a recognizable CM/DIF signal **(A1)** and noise with no visible CM/DIF component **(B1)**, respectively, in response to a 500 Hz stimulus. Their corresponding time frequency scalograms generated using continuous wavelet transformation (CWT) are shown in **(A2,B2)**. These scalograms were then used to train and test the deep learning algorithm. DIF, difference; CM, cochlear microphonic.

Time frequency scalogram images for the classifier were generated from our dataset using CWT and the Python module PyWavelets (44). In this process, a Morlet wavelet shrinks and expands to map the signals into a time-frequency scalogram. We chose the Morlet wavelet because it offers a good compromise between spatial and frequency resolution (33, 45). We normalized the scalograms and compressed them to a dimension of 224 × 224 × 3 for width, height, and depth (red, green, blue). ECochG DIF traces and their wavelet transformation are shown in Figure 3.

We used PyTorch (v 1.11.0) and the pre-trained (on the ImageNet database) AlexNet loaded from torchvision (v 0.6.0) to take advantage of the already good classification properties (46, 47). We substituted the last two classifiers of the AlexNet for binary classification output. The rest of the network was left exactly as it was during initialization. Stochastic gradient descent with momentum was used to train the model. The mini-batch size was 8 and the maximum epoch was 25 with the learning rate being 1e-4, and a momentum of 0.9. We used 10-fold cross-validation to detect overfitting. We then trained the model with the full training set to increase model performance.

### 2.5. Statistical analysis

We used accuracy, sensitivity, and specificity to evaluate our algorithms. The algorithms were compared using the area under the receiver operating characteristic (ROC) curve, also known as the area under the curve (AUC). We used a one-sided DeLong test with a confidence level of 0.95 using the *roc.test* function of the pROC package (v 1.18.0) with R (v 4.1.2) (48, 49).

## 3. Results

### 3.1. ECochG recordings and preprocessing

Gaussian weighted averaging significantly increased the mean SNR from 2.50 dB (standard deviation, SD, 2.39) to 4.18 dB (SD 1.86) as demonstrated by the one-tailed paired-samples *t*-test (*p* < 0.001). In total, 4133 DIF signals were labeled visually by the three experts. Labeling took between 13.5 and 15 h (on average, 12 s per signal). In contrast, objective analysis using the algorithms took less than 25 ms per signal (the duration was determined on a notebook XPS 13 9360 (Dell, Round Rock, TX, USA) and does not include the training time of the algorithms, which was substantially longer).

### 3.2. Visual analysis

The Fleiss' kappa value of the agreement for the examiners and all stimulation frequencies are shown in Table 3. Results demonstrated a substantial to almost perfect agreement among the examiners (50). Particularly, for the mid-frequencies (500 Hz – 1 kHz), the examiners were very much in agreement. This agreement was a little lower for the lowest (i.e., 250 Hz) and the two highest frequencies (i.e., 1,500 and 2,000 Hz), but still substantial. However, between the three examiners, there was a systematic discrepancy in the visual assessment. The false-positive rates (FPRs) for examiners 1, 2, and 3 were 0.110, 0.068, and 0.032, respectively. That is examiner 1 still considered signals with a lot of noise as valid responses, whereas examiner 3 only accepted clearer neurophysiological traces.

**Table 3:** Fleiss' kappa among all three examiners.

**Frequency (Hz)**	**Fleiss' kappa**	**Interpretation**
250	0.748	Substantial agreement
500	0.860	Almost perfect agreement
750	0.868	Almost perfect agreement
1,000	0.858	Almost perfect agreement
1,500	0.799	Substantial agreement
2,000	0.740	Substantial agreement
Mean	**0.815**	Almost perfect agreement

Interpretation according to Landis and Koch et al. (50).

Table 4 shows an overview of the stimulation frequencies, the stimulation levels, the SNR, and the number of signals where the experts identified a CM/DIF response. For frequencies of 500 Hz and above, when stimulated at supra-threshold level, a clear CM/DIF component was found in 53.3%.

**Table 4:** Overview of the stimulation frequencies, the individual intensities, and the SNR.

**Frequency (Hz)**	**Threshold**	***n***	**Ampl (dB)**	**Ampl STD**	**SNR (dB)**	**SNR STD**	***n* visible**	**% n visible**
250	Supra	226	27.33	2.71	2.68	1.34	49	21.7
	Near	222	26.19	2.32	2.32	0.40	10	9.0
	Sub	135	26.50	2.21	2.28	0.27	6	4.4
500	Supra	301	27.61	5.32	4.41	5.55	144	47.8
	Near	283	25.00	2.48	2.62	0.66	43	15.2
	Sub	161	24.50	2.60	2.37	0.39	2	1.2
750	Supra	225	28.00	5.92	4.20	5.30	114	50.1
	Near	272	25.30	3.45	2.80	2.70	63	23.2
	Sub	190	24.92	2.84	2.41	1.17	12	6.3
1,000	Supra	212	29.62	7.88	5.64	6.96	120	56.6
	Near	333	25.24	3.86	2.95	2.83	123	36.9
	Sub	200	24.09	2.60	2.38	0.78	10	5.0
1,500	Supra	193	27.44	6.31	3.98	5.18	102	52.8
	Near	301	24.38	3.11	2.40	1.20	67	22.2
	Sub	187	23.84	3.43	2.30	1.03	8	4.3
2,000	Supra	176	27.35	7.05	4.24	5.39	110	62.5
	Near	270	24.09	3.23	2.40	0.95	81	30.0
	Sub	227	23.23	3.23	2.42	1.02	35	15.4

Frequency, pure tone frequency in Hz; Ampl, peak-to-peak amplitude (dB re 1 μ*V*): SD, standard deviation; SNR, signal-to-noise ratio; n, number of entries. n visible: signals where all examiners indicated a visible cochlear microphonic component.

For all frequencies, the supra-threshold stimulation showed the largest amplitudes (*p* < 0.001, one-tailed paired-samples *t*-test), the biggest SNR (p < 0.001) as well as the most visible signals. Near-threshold stimulation showed larger amplitudes (*p* < 0.001), and bigger SNR (*p* < 0.001) than sub-threshold stimulation. However, this was not the case for 250 Hz stimulation amplitudes (*p* = 0.104). Regarding visual analysis, near-threshold levels showed significantly more visible CM/DIF responses than sub-threshold levels, except at 250 Hz. At this frequency, we identified the same number of responses for near-threshold and sub-threshold levels.

### 3.3. Comparison of objectification methods

**Figure 4:**
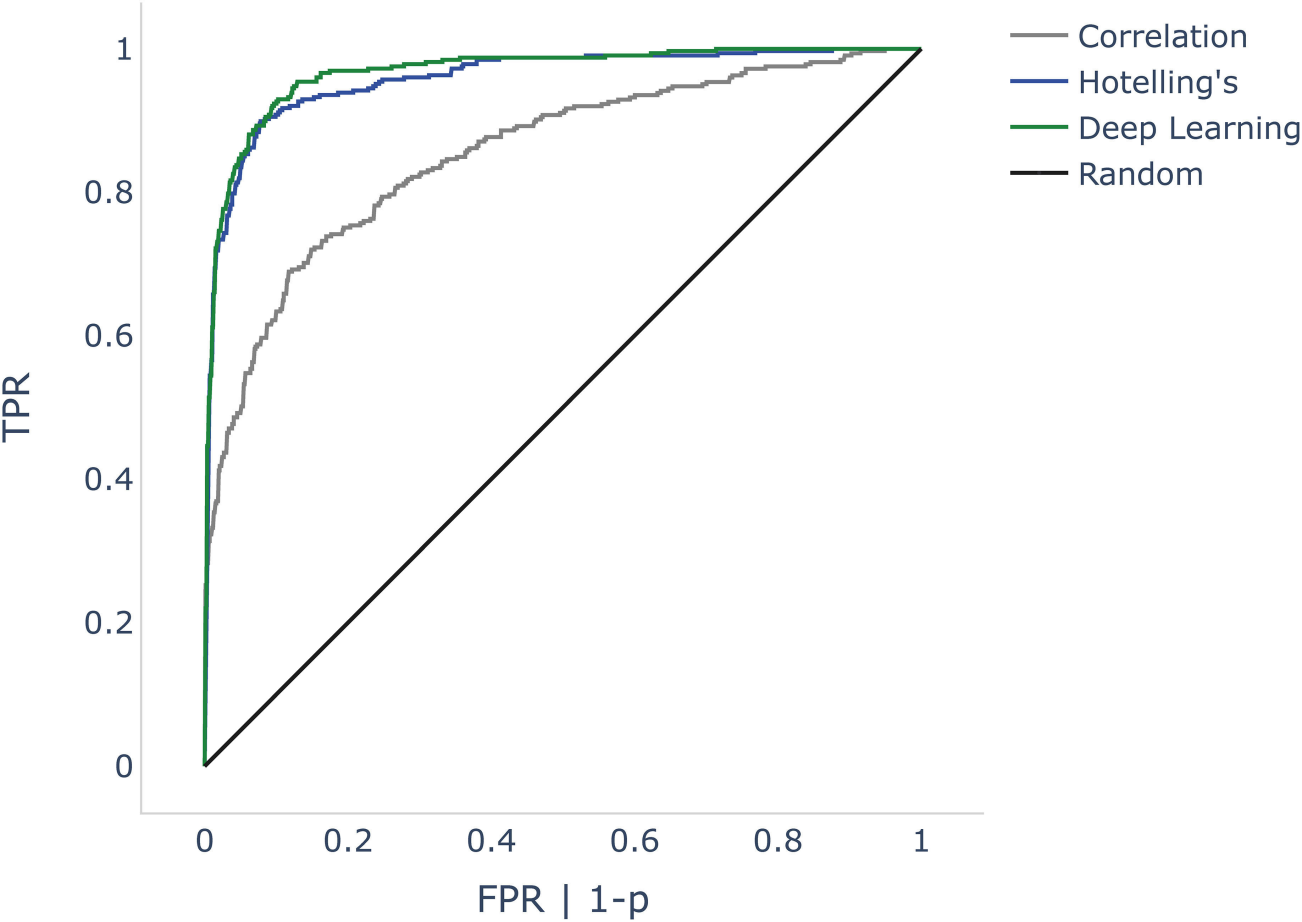
ROC curves comparing correlation analysis, Hotelling's T^2^ test, and deep learning (DL) methods. The false positive rate (FPR) is the dependent variable (x-axis) in the DL and correlation algorithms. As Hotelling's T^2^ test does not specify probabilities, we used *p*-values instead. Since p is inversely proportional to probabilities, we mapped 1-p. The black line shows a random classifier. ROC, receiver operator characteristic; p, *p*-value.

All objectification methods presented in Table 5 showed good performance in detecting CM/DIF responses (51). The ROC curves of the objectification methods for all mixed frequencies are shown in Figure 4. The DL method performed best (AUC = 0.97, accuracy = 92%), followed closely by Hotelling's T^2^ test (AUC = 0.96, accuracy = 91%). Statistically, this difference was not significant (*p* = 0.14). In contrast, the correlation analysis method underperformed as a classifier (AUC = 0.85; accuracy = 83%). This difference was statistically significant (DL *p* < 0.001; Hotelling's T^2^ test *p* < 0.001). Table 5 shows the performance of the algorithms for all frequencies.

**Table 5:** Performance of objectification methods.

**Frequency**	**Correlation analysis**					**Hotelling's T**^**2**^ **test**						**Deep learning**			
**(Hz)**	**Acc**	**Sens**	**Spec**	**CI**	**AUC**	**Acc**	**Sens**	**Spec**	**CI**	**AUC**	**Q**	**Acc**	**Sens**	**Spec**	**CI**	**AUC**
250	0.92	0.19	1	0.04	0.64	0.90	0.83	0.91	0.04	0.96	90	0.96	0.88	0.98	0.03	0.98
500	0.85	0.50	0.99	0.05	0.91	0.93	0.95	0.92	0.03	0.97	80	0.92	0.94	0.91	0.04	0.97
750	0.84	0.50	0.98	0.05	0.88	0.93	0.91	0.93	0.04	0.98	100	0.94	0.91	0.95	0.03	0.97
1,000	0.81	0.58	0.94	0.05	0.84	0.86	0.95	0.82	0.05	0.97	85	0.91	0.88	0.92	0.04	0.97
1500	0.82	0.42	0.97	0.05	0.82	0.95	0.95	0.94	0.03	0.99	105	0.93	0.95	0.92	0.04	0.99
2,000	0.77	0.44	0.95	0.06	0.81	0.89	0.78	0.96	0.05	0.91	100	0.84	0.71	0.92	0.06	0.92
all	0.83	0.52	0.95	0.02	0.85	0.91	0.91	0.91	0.02	0.96		0.92	0.88	0.94	0.02	0.97

Q is the number of TVMs used in Hotelling's T^2^ test. The optimal number of TVMs depends on the frequency and is given in the Q column. Stim, stimulus frequency (Hz); Acc, accuracy; Sens, sensitivity; Spec, specificity; CI, 95% confidence interval; AUC, area under the receiver operator characteristic curve; Q, number of used TVMs for Hotelling's T^2^ test.

## 4. Discussion

This study demonstrates that it is possible to objectively and automatically determine whether a CM/DIF response is present or not. All three algorithms investigated showed very good to excellent discrimination performance. Especially Hotelling's T^2^ test and the DL method revealed excellent results (mean accuracy was 91 and 92% with an AUC of 0.96 and 0.97, respectively).

### 4.1. Preprocessing

ECochG traces are usually displayed as averaged signals (both, intra,- and postoperatively). During signal recordings, noisy epochs can affect the signal quality and reduce SNR (34). In addition, there are large inter-individual differences. While some patients show very prominent potentials, in others the signal amplitude is small (1, 3, 10, 12, 52). If ECochG is to be used routinely in the operating room and postoperative setting, however, all patients (including those with small signals) must be analyzed. In our cohort, the previously described Gaussian weighted averaging method (34, 35) showed a substantial increase in SNR of ECochG signals of all frequencies. Our calculations improved the mean SNR by 1.68 dB. Kumarange et al. were able to improve the SNR by 3.5 dB. However, they used extracochlear ECochG recordings, whereas we measured from inside the cochlea.

### 4.2. Visual analysis

In our study, the visual evaluation of the data was carried out by three independent examiners who have many years of experience in this field. Per recording, it took them 12 s on average to judge if a signal was present or not. In contrast, with the described computer algorithms, the evaluation was available after a few milliseconds. This time span may not sound like much. But it is crucial, especially in the intraoperative real-time setting, where immediate decisions must be made to prevent possible inner ear injury.

Regarding the visual analysis, the agreement of the three examiners was very good, especially in the frequency range between 500 and 1,000 Hz. Disagreements occurred mainly in borderline cases with low SNRs (another reason why the SNR needs to be improved, if possible). The agreement among the experts was still substantial, but lower 250 Hz and for the two highest frequencies (i.e., 1,500 and 2,000 Hz). At 250 Hz, among all measured intracochlear ECochG, the SNR was the lowest (also refer to Table 4) (8). For the two highest frequencies, in some cases, it was difficult to distinguish between natural signal fluctuations and reproducible CM/DIF signal components.

It is important to note that a low SNR can affect the waveform morphology. In our data, e.g., CON and RAR responses did not evolve in opposite directions to each other, or there was a change in the usual morphology (e.g., the characteristic frequency of the CM/DIF signal was too low, or the ECochG traces had an irregular shape). This resulted in one examiner detecting a CM/DIF response while the other detected only noise. In our analysis, we found that the overall agreement was high, but one expert was rather cautious and another more tolerant in his assessment. This issue can be addressed by using an automated, quantitative and objective evaluation method, as suggested by our study. This allows for a uniform evaluation of the signals, which simplifies the comparison between individuals and different implantation centers or even makes it possible in the first place.

The analysis of the three stimulation levels showed that supra-threshold stimulation most frequently elicited a visually present CM/DIF signal. In addition, the SNR (except at 250 Hz) was substantially higher compared to the near- and sub-threshold levels. With supra-threshold stimulation, in our cohort, for the frequencies 500 Hz and above, a clear CM/DIF response was detected in 53.3% of cases. This implies that in a significant proportion of cases, no clear response could be detected. Additionally, this is despite the fact that most of the measurements took place in a postoperative setting and patients had a measurable residual hearing on the day of examination. However, it should be noted that the PTA of our study population shows a large variance and was, in some individuals, above 90 dB (compare Table 1). Consequently, the stimulus intensity was not always equally above the hearing threshold. In addition, recordings were measured from 4 different electrodes. For many subjects, ECochG responses were not visible at all electrodes. In literature, the situation regarding the prevalence of CM/DIF responses when stimulating above the hearing threshold is controversial. While some authors have found a close correlation between hearing threshold and CM/DIF signal threshold (11), other scientists have not found a clear relationship (1, 2, 8, 9, 12, 22, 23). Based on our data (refer to Table 4), we must assume that this correlation is both level- and frequency-dependent. For near-threshold and sub-threshold simulations, we detected significantly fewer visually detectable ECochG signals. Interestingly, the sub-threshold stimulation also showed CM/DIF responses in some cases (9, 23). Especially at 2,000 Hz, this finding was more pronounced.

### 4.3. Comparison of the objectification methods

In our study, DL with CNN AlexNet on time-frequency scalogram plots using CWT showed the best discrimination performance. The advantage of this method is that the morphology of the electrophysiological signal is taken into account. Similar to visual inspection, our algorithm was able to identify the CM/DIF response in the time-frequency scalograms shown in Figure 3. Another advantage of DL is its independence from preprocessing steps of ECochG signals (e.g., filtering). We trained our network with both, filtered and unfiltered data and could observe an almost identical accuracy of 90%.

Hotelling's T^2^ test showed the highest sensitivity of our tested algorithms. This high sensitivity is also known from other research (27–29). However, in order to achieve good results with the Hotelling T^2^ method, the signal must be free of artifacts and baseline wander. Both signal phenomena occur in ECochG measurements and must be addressed by using preprocessing steps. Furthermore, an optimal length of the TVMs must be defined. This is a trade-off; if the TVMs are too long, they contain the natural fluctuation of the ECochG signal (e.g., peaks and valleys). This results in TVMs with zero amplitude (similar to noise). If the TVMs are too short, the robustness and thus the test sensitivity decreases (overfitting) (29, 39).

Finally, the correlation analysis gave good objectivity to our data, although it did not reach the performance of the other two methods. It should be noted that signal artifacts can also have a high correlation and thus reduce the accuracy of this method. Such artifacts arise, e.g., from stitching or other unwanted effects (25). To overcome this, one could try to eliminate artifacts with more elaborate techniques or correlate only segments that are not affected.

In summary, the DL algorithm and Hotelling's T^2^ test are very well suited for the objective assessment of ECochG signals; we achieved a high accuracy with both approaches. By using one of these methods, we can evaluate CM/DIF signals independently of the expertise of the examiner. In this article, we focused on the methodology itself with the question of whether a CM/DIF response was present or not. In the next step, further calculations could be included. For example, the evolution of amplitude or latency during electrode insertion. Furthermore, the advantages of the methodology are the immediate result as well as the reproducibility, which allows the comparison i) between individuals, ii) between different implant centers as well as iii) of longitudinal data. Finally, an automated ECochG assessment tool would pave the way for future standardized and widespread use in the clinical setting.

### 4.4. Limitations

Our data set was limited to 4133 ECochG recordings. Additional signals would further improve the methodology, increase the generalization of our models and reduce overfitting. Moreover, the data were visually reviewed by three experts. If more experts were incorporated into the algorithm, this may also refine the evaluation. Systemic noise can hamper the use of objective algorithms. In particular, the correlation analysis and Hotelling's T^2^ test were found to be vulnerable. The DL method on the other hand was less dependent on data preprocessing and less sensitive to noise interference.

We have applied our methodology only when the electrode position was stable. In the next step, the objectification methods must also be tested during insertion, i.e., when the electrode is in motion. Furthermore, in the current study, we restricted ourselves to the CM/DIF signal. However, the methodology could also be used for the other signal components (i.e., ANN/SUM, CAP, SP). The combination of different data features is also advisable (4, 53) and must be evaluated in a future study.

## 5. Conclusion

Objectification of ECochG signals is possible with the methods presented in this paper. Our DL algorithm and Hotelling's T^2^ test achieved a high accuracy to detect CM/DIF responses that had previously been identified by three ECochG experts. Objective automatic analysis of CM/DIF signals enables standardized, fast, accurate, and examiner-independent evaluation of ECochG measurements.

## Data availability statement

The raw data supporting the conclusions of this article will be provided by the authors upon request.

## Ethics statement

The studies involving human participants were reviewed and approved by the Cantonal Ethics Committee of Bern (BASEC ID 2019-01578). Written informed consent to participate in this study was provided by the participants' legal guardian/next of kin.

## Author contributions

KS performed the measurement, wrote the software and article, and labeled and analyzed the data. WW analyzed the data and provided interpretive analysis and critical revision. AD labeled the data. CR, MC, and GM provided interpretive analysis and critical revision. SW designed the experiment, analyzed the data, labeled the data, and provided interpretive analysis and critical revision. All authors contributed to the article and approved the submitted version.

## Funding

This study was partly funded by the Department of Otorhinolaryngology, Head and Neck Surgery at the Inselspital Bern, the Clinical trials unit (CTU) research grant, and the MED-EL company. GM was supported by the Swiss National Science Foundation #320030_173081. The authors declare that this study received funding from MED-EL Germany. The funder was not involved in the study design, collection, analysis, interpretation of data, the writing of this article or the decision to submit it for publication.

## Conflict of interest

The authors declare that the research was conducted in the absence of any commercial or financial relationships that could be construed as a potential conflict of interest.

## Publisher's note

All claims expressed in this article are solely those of the authors and do not necessarily represent those of their affiliated organizations, or those of the publisher, the editors and the reviewers. Any product that may be evaluated in this article, or claim that may be made by its manufacturer, is not guaranteed or endorsed by the publisher.
