# Supplementary material for: Objectification of intracochlear electrocochleography using machine learning
Source: Front Neurol. 2022 Aug 29;13:943816. doi: 10.3389/fneur.2022.943816 (PMC9465334; doi:10.3389/fneur.2022.943816)
Supplement: Supplementary file 1 [file Data_Sheet_1.PDF]

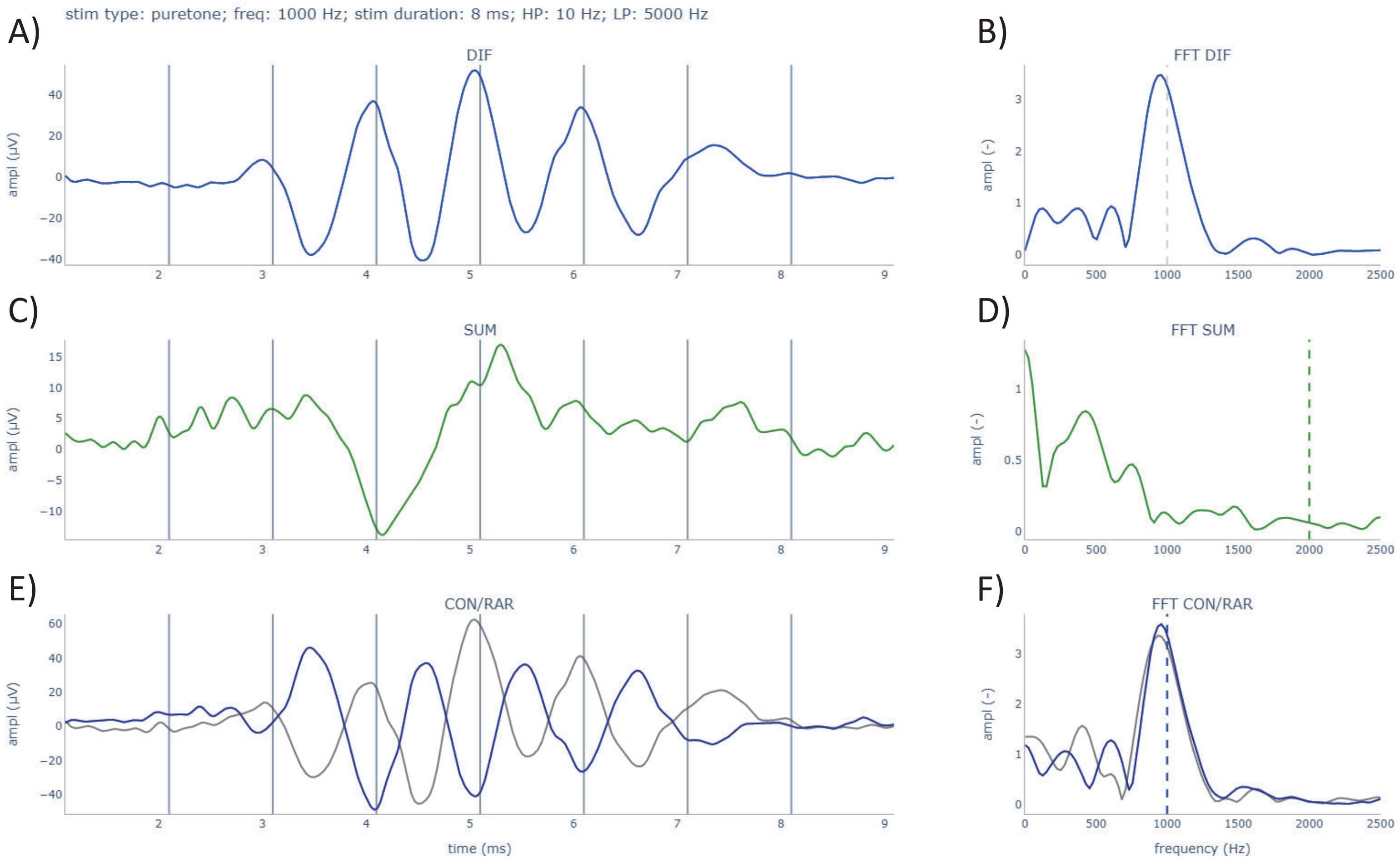

For the visual analysis, the three experts were presented with the following subplots for each ECochG signal: A) DIF response, C) SUM response, E) CON and RAR responses, and B, D, F) their individual FFT traces. The gray vertical lines indicate the stimulus period. The dashed vertical lines indicate the expected frequency of the response.
